# Association between hair cortisol levels and keratoconus: A prospective study

**DOI:** 10.1371/journal.pone.0331670

**Published:** 2025-09-12

**Authors:** Noor Alqudah, Nosayba Al-Azzam, Leen El Taani, Mohammad Al Qudah, Abdallah Sharayah, Rita A. Al-Rihani

**Affiliations:** 1 Department of Special Surgery, Division of Ophthalmology, Faculty of Medicine, Jordan University of Science and Technology, Irbid, Jordan; 2 Department of Physiology and Biochemistry, Faculty of Medicine, Jordan University of Science and Technology, Irbid, Jordan; 3 Department of Neurology, Faculty of Medicine, Jordan University of Science and Technology, Irbid, Jordan; Democritus University of Thrace, GREECE

## Abstract

**Introduction:**

Keratoconus is a progressive corneal disorder characterized by corneal thinning often leading to significant visual impairment. Recent research suggests a potential role of cortisol in the development and progression of keratoconus. However, the relationship between cortisol levels and keratoconus severity remains poorly understood.

**Purpose:**

To investigate the association between hair cortisol levels and keratoconus severity in a Jordanian population, exploring whether cortisol could serve as a potential biomarker for disease progression or stability.

**Methods:**

We conducted a prospective, observational study at King Abdullah University Hospital in Jordan. Sixty participants aged 18–30 years were categorized into three groups: Healthy (n = 20), Progressive Keratoconus (n = 20), and Stable Keratoconus (n = 20). Demographic, clinical, and ocular data were collected, including age, gender, hair cortisol levels, and corneal topography indices (Kmax, Kmean, and thinnest corneal location).

**Results:**

The median cortisol level was significantly higher in the Progressive Keratoconus group (987 pg/mL) compared to the Stable group (300 pg/mL, p-value <0.001). Regression analysis revealed a significant negative association between cortisol levels and stable keratoconus (β = −1.31, p < 0.001). No significant association was observed for progressive keratoconus after adjustment for age and gender. Correlation analysis showed no significant associations between cortisol levels and BMI, Kmax, or Kmean. Cortisol levels also did not vary significantly across keratoconus severity categories in either eye.

**Conclusion:**

Elevated cortisol levels may be associated with keratoconus progression, while lower levels appear linked to disease stability. Cortisol could serve as a potential biomarker for disease monitoring; however, further multicenter studies are needed to validate these findings and clarify cortisol’s role in keratoconus pathogenesis.

## Introduction

Keratoconus is a progressive condition characterized by the abnormal steepening of the cornea, and it is recognized as one of the primary causes of corneal transplants. Keratoconus typically manifests in young adults and adolescents with irregular astigmatism and decreased vision [1,2]. There is a global disparity in the prevalence of keratoconus based on geographic location, diagnostic criteria, and the characteristics of the studied population [3,4]. In the Middle East, a high prevalence was noted, reaching 2.34% [5,6]. In Jordan, a hospital-based study by Al-Bdour et al. showed that keratoconus was identified as the cause of 5% unilateral and bilateral visual loss in patients over the age of 20 [7].

Keratoconus is linked to several risk factors, such as chronic eye rubbing, prolonged use of rigid gas-permeable contact lenses, ultraviolet light exposure, and conditions like atopy [8]. It is also associated with systemic disorders, including Down syndrome, Leber’s congenital amaurosis, and connective tissue diseases [9]. The condition is considered hereditary, with a positive family history observed in 6% to 8% of cases, although environmental factors also contribute to its progression [10].

Environmental factors are the most acceptable etiology due to the multifactorial nature influencing the development of keratoconus. In addition, environmental factors are crucial in triggering keratoconus in patients with a genetic predisposition to keratoconus [11]. The rationale behind their role in developing keratoconus stems from the oxidative damage to keratoconus corneas and the production of reactive oxygen species (ROS). In addition to enzymatic deficiencies, this ultimately leads to corneal thinning and vision loss [11,12].

Studies have indicated that collagenase enzymes, including matrix metalloproteinases (MMPs), may play a role in the pathogenesis of keratoconus. These MMPs are found to be upregulated in relation to the disruption of the cortisol rhythm [13].

Acute cortisol measurements from blood, saliva, or urine samples can be highly variable due to significant ultradian and circadian fluctuations and the influence of various situational factors [14]. Measurement of cortisol through hair samples serves as a reliable biomarker for monitoring chronic stress and exposure to specific drugs and offers greater stability and reduced dependence on circadian rhythms compared to blood or urine measurements. This method provides more consistent and accurate insights into long-term cortisol patterns and their association with keratoconus progression [14,15]. In addition, hair cortisol levels demonstrate intra-individual stability for at least four months, making them a reliable indicator of basal cortisol levels over extended periods. Unlike daily cortisol fluctuations, hair cortisol reflects long-term cortisol production more effectively, capturing trends over one to four months rather than short-term, day-to-day variations [16].

Cortisol may play a significant role in the pathogenesis of keratoconus. However, to date, no published in vivo evidence has demonstrated that elevated cortisol levels directly influence the progression of the disease. Therefore, in this prospective, single-center study, we aim to evaluate the association between cortisol levels and keratoconus severity and stability in Jordanian population.

## Methods

### Study design and patient population

We conducted a prospective, observational at the Ophthalmology Department of King Abdullah University Hospital from March 2024 to October 2024. The study adhered to the tenets of the Declaration of Helsinki and was approved by our hospital’s Institutional Review Board (IRB: 6/166/2024). Before the examination, a written consent form was obtained from each participant.

A total of 60 participants from whom hair samples were collected for this study, were recruited from our clinics and aged between 18 and 30 years, were categorized into three groups: healthy controls (n = 20), progressive keratoconus (n = 20), and stable keratoconus (n = 20).

Ethical approval was obtained from the Institutional Review Board of Jordan University of Science and Technology with the ref number (**6/166/2024**).

Inclusion criteria for the progressive keratoconus group were patients who were diagnosed with keratoconus and showed significant changes in corneal topographic parameters, as determined through corneal topographic analysis using a Pentacam device (Oculus Optikgeräte, Wetzlar, Germany). Significant progression of keratoconus was defined as an increase in the maximum keratometry value at the corneal apex by 1.00 diopter (D) within one year and/or a mean decrease in corneal thickness by 5% or more over the past 12 months. The stable keratoconus group included patients with keratoconus who had undergone corneal cross-linking (CXL) at least one year prior to the study and showed no progression based on the aforementioned criteria.

Participants were excluded for the following reasons: (1) the presence of other corneal pathologies, (2) a history of ocular surgery, uveitis, glaucoma, connective tissue disease, rheumatological disorders, diabetes mellitus, or psychiatric diseases, (3) a history of medications that might affect cortisol levels, including systemic corticosteroids, selective serotonin reuptake inhibitors (SSRIs), tricyclic antidepressants, anxiolytics, and inhaled corticosteroids for asthma; (4) presence of systemic disorders known to affect cortisol metabolism, such as hypertension and thyroid dysfunction (5) and pregnancy.

### Hair sample collection and preparation

Hair samples were collected by trained ophthalmology residents. Each sample, consisting of 150–200 strands, was taken from the posterior vertex of the scalp, ensuring proximity to the scalp to include the proximal region. For female patients, only the proximal 3 cm of hair was retained, while for male patients, the entire strands were preserved without further cutting. Samples were stored at room temperature until analysis. Hair strands were carefully cut using sterilized surgical scissors, and approximately 35 mg of hair was weighed and transferred into a clean Eppendorf tube. To eliminate external contaminants, 1.5 mL of hexane was added to each tube, and samples were incubated at room temperature for 2 minutes. The hexane was then carefully removed, and the hair was dried under a gentle nitrogen gas stream. The dried samples were flash-frozen using liquid nitrogen to preserve integrity.

The frozen hair samples were then ground into a fine powder using a homogenizer, with 30-second pauses between cycles to prevent overheating. Subsequently, 1.8 mL of methanol was added, and the samples were thoroughly vortexed. The tubes were placed on a shaker overnight at room temperature to ensure complete cortisol extraction. The methanol extract was transferred to a clean tube and evaporated under a gentle air stream at 37°C. The remaining residue was reconstituted in 400 μL of ELISA Buffer (1X, Cat. No: 400060) and stored at −80°C until analysis.

Cortisol concentrations were quantified using an enzyme-linked immunosorbent assay (ELISA). The assay was performed with a commercially available ELISA kit (Cat. No: 500360) purchased from Cayman (Ann Arbor, Michigan, USA), following the manufacturer’s instructions.

### Data collection

Demographic and clinical data were collected for demographic and clinical variables and included age, gender, body-mass index (BMI), occupational status, smoking history, and medical history. Ocular data included baseline ophthalmic assessments such as corneal topography indices (Kmax – maximum keratometry, Kmean and thinnest corneal location). For the Progressive and Stable Keratoconus groups, disease progression status and history of cross-linking procedures were also recorded. Based on existing literature, hair cortisol levels in healthy adults typically range from ~5.1–459.6 pg/mL, although values may vary depending on population differences [14]. The severity of keratoconus in each eye was classified as mild (Kmean <48), moderate (Kmean ≥48 and <53), and severe (Kmean ≥53).

### Statistical analysis

To evaluate the adequacy of our sample size, we conducted an a priori power analysis using the pwr.anova.test() function in R. Based on previously reported and observed means and standard deviations of hair cortisol levels among the three groups—progressive keratoconus (M = 624 pg/mg, SD = 160), stable keratoconus (M = 368 pg/mg, SD = 64.7), and healthy controls (M = 351 pg/mg, SD = 89.6)—we calculated a large effect size (Cohen’s f = 1.36) [17]. Using this effect size, with a significance level (α) of 0.05 and a desired power (1–β) of 0.80, the estimated minimum required sample size was 3 participants per group. Our study included substantially larger group sizes, supporting the adequacy of statistical power to detect group differences in cortisol levels.

Descriptive statistics were applied to summarize demographic and clinical characteristics within each group. Continuous variables were reported as medians with interquartile ranges (IQR), while categorical variables were expressed as percentages. The normality of data distribution was assessed using the Shapiro-Wilk test. Group differences were compared using correlation coefficients and linear regression models for continuous variables. A p-value <0.05 was considered statistically significant for any exploratory analysis. Results are presented in tables and figures where appropriate.

## Results

A total of 60 patients, with a median age of 24.0 (20.8, 27.3) years with even gender. The median BMI was 25.3 kg/m² (23.4, 29.5). Among the participants, 37% were smokers. Occupational status varied, with 38% employed, 3.3% self-employed, 42% students, and 17% unemployed. The median cortisol level was 438 (288, 994) pg/ml (Table 1). Median age was significantly different among the groups (p = 0.005), with stable keratoconus patients being older (26.5 years) compared to progressive keratoconus (22.0 years) and healthy participants (24.0 years. Occupational status also varied significantly (p-value = 0.012), with students dominating the progressive keratoconus group (55%) and employed participants in the majority of the stable keratoconus group (45%) as shown in Table 1.

**Table 1:** Comparison between demographic and clinical variables across study groups.

Characteristic	Healthy N = 20	Progressive Keratoconus N = 20	Stable Keratoconus N = 20	p-value	Overall N = 60
**Age (Years)**	24.0 (19.0, 27.3)	22.0 (19.0, 25.0)	26.5 (24.0, 30.0)	0.005	24.0 (20.8, 27.3)
**Gender**				1.0	
Female	10 (50%)	10 (50%)	10 (50%)		30 (50%)
Male	10 (50%)	10 (50%)	10 (50%)		30 (50%)
**BMI (Kg/m**^**2**^)	25.2 (23.3, 28.9)	24.6 (23.1, 26.1)	29.0 (24.3, 34.9)	0.077	25.3 (23.4, 29.5)
**Smoking**	9 (45%)	7 (35%)	6 (30%)	0.6	22 (37%)
**Occupation**					
Employed	10 (50%)	4 (20%)	9 (45%)		23 (38%)
Self-employed	0 (0%)	1 (5.0%)	1 (5.0%)		2 (3.3%)
Student	10 (50%)	11 (55%)	4 (20%)		25 (42%)
Unemployed	0 (0%)	4 (20%)	6 (30%)		10 (17%)
**Cortisol level (pg/ml)**	658 (410, 1,015)	987 (414, 1,398)	300 (211, 366)	**<0.001**	438 (288, 994)

Cortisol levels were significantly different (p-value < 0.001), with progressive keratoconus patients having the highest median levels (987 pg/ml) and stable keratoconus patients the lowest (300 pg/ml). Pairwise comparison analysis showed a significant difference between stable and progressive keratoconus patients, with higher cortisol levels in progressive keratoconus (mean difference: 735.0, p-value <0.001, Table 2), while no difference was seen between healthy and stable keratoconus, and between healthy and progressive keratoconus.

**Table 2:** Pairwise comparison of cortisol levels across study groups.

Contrast	Mean difference (SE)	Adjusted p-value
Healthy – Progressive keratoconus	−289.43 (190.1)	0.406
Healthy – Stable keratoconus	445.61 (190.9)	0.070
Progressive keratoconus – Stable keratoconus	735.04 (183.0)	<0.001

In the linear regression model adjusted for age, occupation status grouped as employed and unemployed, and gender, when comparing the healthy group to the progressive keratoconus group, no significant association with cortisol levels was found (p-value = 0.115), however, the comparison between the stable keratoconus group and the progressive keratoconus group revealed a significant negative association with cortisol levels (p-value <0.001) which means that patients with stable keratoconus tended to have significantly lower cortisol levels compared to those with progressive keratoconus as shown in Table 3.

**Table 3:** A linear regression model between cortisol levels and study variables adjusted for age, occupation, and gender.

		95% Confidence Interval		
**Predictor**	**Stand. Estimate**	**Lower**	**Upper**	**p-value**
**Age**	0.183	−0.09	0.46	0.199
**Group:**				
Healthy – Progressive Keratoconus	−0.519	−1.13	0.09	0.094
Stable Keratoconus – Progressive Keratoconus	−1.29	−1.93	−0.64	< .001
**Gender:**				
Male – Female	−0.324	−0.84	0.19	0.216
**BMI**	0.003	−0.26	0.27	0.982
**Occupation:**				
Unemployed – Employed	−0.265	−0.98	0.45	0.464

Spearman’s correlation analysis revealed no significant associations between cortisol levels and BMI (R = −0.068, p = 0.617), K-max (R = −0.039, p = 0.810), or K-mean (R = −0.104, p = 0.523) in the more severe eye for each patient. However, there was a trend towards a positive correlation between cortisol levels and the thinnest corneal location (R = 0.290, p = 0.069), although it did not reach statistical significance (Table 4).

**Table 4:** Correlation between cortisol levels and keratometry parameters in the more severe eye for each patient.

Parameter	Spearman’s R	P-value
**K-max**	−0.039	0.810
**K-mean**	−0.104	0.523
**Thinnest location**	0.290	0.069

* More severe eye for each patient was decided according to higher values of K mean, k max and lower thinnest location compared to the other eye in same patient.

When comparing cortisol levels and different classifications of keratoconus in each eye for the same patient, no statistically significant differences were observed in cortisol levels across mild, moderate, and severe cases in both the right (OD) and left (OS) eyes. In the right eye, cortisol levels were highest in severe keratoconus (455 pg/ml), followed by mild (398 pg/ml), and lowest in moderate cases (262 pg/ml) (p = 0.5). In the left eye, cortisol levels remained relatively stable across severity groups: mild (365 pg/ml), moderate (675 pg/ml), and severe (455 pg/ml) (p = 0.4) as shown in Table 5, which may indicate limited association between cortisol levels and disease severity based on laterality.

**Table 5:** Comparison of cortisol levels and severity of keratoconus in each eye.

OD:				
Characteristic	Mild, N = 31	Moderate, N = 7	Severe, N = 2	p-value
**Cortisol (pg/ml)**	398 (264, 1,005)	262 (221, 484)	455 (439, 470)	0.5
**Age (Years)**	24.0 (21.0, 26.0)	24.0 (21.5, 30.0)	24.0 (21.0, 27.0)	0.8
**Gender**				0.5
Female	15 (48%)	3 (43%)	2 (100%)	
Male	16 (52%)	4 (54%)	0 (0%)	
**OS:**				
**Characteristic**	**Mild, N = 31**	**Moderate, N = 7**	**Severe, N = 2**	**p-value**
**Cortisol (pg/ml)**	365 (211, 934)	675 (273, 1,616)	455 (439, 470)	0.4
**Age (Years)**	24.0 (21.0, 26.5)	26.0 (24.0, 28.0)	24.0 (21.0, 27.0)	0.6
**Gender**				0.4
Female	14 (45%)	4 (57%)	2 (100%)	
Male	17 (55%)	3 (43%)	0 (0%)	

## Discussion

Keratoconus is a progressive corneal disease characterized by corneal thinning and irregular astigmatism, leading to visual impairment [18]. Its prevalence varies significantly across regions, with high rates reported in the Middle East, including Saudi Arabia and Jordan [5]. Environmental factors, alongside genetic predispositions, are believed to play a significant role in keratoconus pathogenesis, with oxidative damage and reactive oxygen species production being key contributors [19]. Cortisol, a stress-related hormone, has been proposed as a potential factor in disease progression, though its exact role remains unclear [20]. In this prospective cohort study, we included 60 participants categorized into healthy presenting controls, progressive keratoconus, and stable keratoconus groups and investigated the potential link between cortisol levels obtained from hair samples and keratoconus to provide insight into whether cortisol could serve as a biomarker for disease stability or severity.

### The main findings of the study

Our study revealed significant difference in hair cortisol levels between keratoconus groups, with the higher levels observed in the progressive keratoconus (987 pg/ml) group and the lower level in stable keratoconus (300 pg/ml) This suggests that cortisol levels may play a role in distinguishing between stable and progressive cases of keratoconus. The regression model indicated a significant negative association between cortisol levels and stable keratoconus, independent of age and gender, which means lower levels of cortisol are associated with stable keratoconus. We did not observe significant associations in the progressive keratoconus group and healthy controls in the regression model after accounting for age and gender. In addition, our study revealed that corneal parameters in the more severe eye for each patient were not correlated with cortisol levels, and the severity of keratoconus was not associated with cortisol levels in both eyes.

In a prospective study by Stival et al. in Brazil investigated the differences between stable and progressive keratoconus in hair cortisol levels, showing that progressive keratoconus had a significantly higher cortisol levels compared to both stable keratoconus and healthy controls. In addition, they showed a significant correlation between cortisol levels and Kmax [21]. Previous studies have shown a potential role of systemic stress markers, including cortisol, in ocular diseases [22]. Elevated cortisol levels have been associated with oxidative stress and tissue degradation; mechanisms also observed in keratoconus pathogenesis. However, our study contrasts with prior findings by suggesting that stable keratoconus patients exhibit significantly lower cortisol levels. This discrepancy highlights the need for further research to clarify cortisol’s role and its potential as a biomarker. However, our findings were consistent with findings from Lenk et al. study showing that no difference was noted between healthy controls and stable keratoconus patients [23]. This could suggest that the association of cortisol and keratoconus is mostly associated with disease progression which was noted by the significantly higher levels in progressive keratoconus patients compared to stable keratoconus patients. In Shetty et al. study they showed that inflammatory cytokines including metalloproteinase-9, interleukin-6, and tumor necrosis factor-α were significantly overexpressed in keratoconus patients and were significantly reduced after treatment with cyclosporine A with a subsequent reduction in corneal curvature [24]. Progressive keratoconus with marked elevation of inflammatory cytokines such as hair cortisol concentration suggest that upregulation of matrix metalloproteinases produced by the corneal epithelium is a key factor responsible for collagen breakdown, therefore, higher cortisol levels correlates with higher metalloproteinases activity, thereby exacerbating the structural damage observed in progressive keratoconus patients [17]. However, the use of metalloproteinases as a biomarker might not be always feasible because its expression is not consistently continuous, making cortisol levels a more reliable biomarker for monitoring keratoconus progression.

While our study showed no difference in hair cortisol levels between progressive keratoconus and healthy controls, this could be attributed to several factors. Cortisol is a systemic stress hormone, and its levels may not fully reflect localized changes in the corneal tissue. Keratoconus progression may involve localized tissue responses that are not directly influenced by systemic cortisol levels [15]. In addition, due to the small sample size, there may be significant inter-individual variability in cortisol responses to stress, making it challenging to detect differences in small sample sizes.

The observation that stable KC patients exhibited significantly lower hair cortisol concentrations than healthy controls may be explained by a combination of biological and psychosocial mechanisms [24]. This observation could be attributed to the long-term anti-inflammatory effects of CXL, which all patients in the stable group had undergone at least one year prior to the study. Previous research has demonstrated that CXL leads to sustained suppression of pro-inflammatory cytokines such as interleukin-1β, interleukin-6, tumor necrosis factor-α, and matrix metalloproteinase-9, beginning within the first three months post-procedure and persisting beyond one year [24].

Also, demographic and psychosocial factors may have contributed to the lower HCC in the stable KC group. These patients were significantly older and more likely to be employed, which is associated with lower perceived stress and cortisol levels compared to younger individuals or students [25]. By contrast, the healthy control group included a higher proportion of university students—a population known to have elevated HCC due to academic pressure and transitional life stressors [26]. Although body mass index (BMI) was higher in the stable group, existing literature on the relationship between BMI and HCC remains inconclusive. Furthermore, while the study controlled major confounders, it did not assess hair treatment practices, ultraviolet exposure, or hair washing frequency, all of which can affect cortisol measurement accuracy. Given the wide inter-individual variability in HCC and the limited sample size, these findings should be interpreted with caution and validated in larger, longitudinal cohorts.

### Clinical implications

Our results suggest that cortisol levels may serve as a potential indicator of disease stability in keratoconus patients. Monitoring cortisol levels could aid clinicians in differentiating between stable and progressive cases, improving early intervention strategies. Increased cortisol levels may contribute to the progression of keratoconus by affecting corneal thickness and biomechanics. Additionally, stress management interventions might be considered as adjunctive therapy for keratoconus management.

### Strengths and limitations

A key strength of the study lies in its novel focus on cortisol levels measured from hair samples, providing long-term hormonal data rather than acute fluctuations. However, limitations include the absence of a standardized stress assessment tool (such as the Perceived Stress Scale (PSS) or the State-Trait Anxiety Inventory (STAI), which may have provided complementary information to the biological measures of stress. In addition, the Single-center design, small sample size, and the lack of longitudinal follow-up, which restricts causal inference, and might limit generalizability of our findings.

### Future directions

Future studies are needed with a multi-center design, larger sample sizes and longitudinal follow-up to validate our findings. Using validated questionnaires such as the Perceived Stress Scale (PSS) or the State-Trait Anxiety Inventory (STAI), to assess stress levels at the time of the study to provide a more comprehensive analysis of this potential confounding factor. It is also crucial to investigate the molecular mechanisms linking cortisol and corneal changes could further elucidate cortisol’s role in keratoconus progression.

## Conclusion

Our study highlights a potential association between cortisol levels and keratoconus progression. While elevated cortisol levels were not directly linked to disease severity, their lower levels in stable cases suggest a possible role in disease modulation and significant stress and oxidative damage in progressive cases. Further research is essential to confirm these findings and explore cortisol as a biomarker for keratoconus management.

## Supporting information

S1 Data Clinical and demographic data of patients with keratoconus and healthy controls. (XLSX)
